# Supplementary material for: Smooth Interpolating Curves with Local Control and Monotone Alternating Curvature
Source: Comput Graph Forum. 2022 Oct 6;41(5):25–38. doi: 10.1111/cgf.14600 (PMC9827861; doi:10.1111/cgf.14600)
Supplement: Supplementary file 1 — Supplement Material [file CGF-41-25-s001.zip › Local-Smooth-Interpolating-MonoCurvature/extern/clothoids/submodules/Utils/ThirdParties/fmt/fmt-8.1.1.zip!/fmt-8.1.1/doc/html/search.html]

Search — fmt 8.1.1 documentation


Toggle navigation

{fmt}

- 8.1.1
  - 8.1.1
  - 8.1.0
  - 8.0.1
- Contents
- Usage
- API
- Syntax

# Search

Please activate JavaScript to enable the search
functionality.

From here you can search these documents. Enter your search
words into the box below and click "search". Note that the search
function will automatically search for all of the words. Pages
containing fewer words won't appear in the result list.

© Copyright 2012-present, Victor Zverovich.
Created using Sphinx 3.3.0.
